# Retraction: Long noncoding RNA SNHG1 activates autophagy and promotes cell invasion in bladder cancer

**DOI:** 10.3389/fonc.2023.1228367

**Published:** 2023-06-12

**Authors:** Frontiers Editorial Office

“The Publisher retracts the cited article. Following publication, concerns were raised regarding the integrity of the images in the published figures. The authors failed to provide a satisfactory explanation during the investigation, which was conducted in accordance with Frontiers’ policies.

This retraction was approved by the Chief Editors of Frontiers in Oncology and the Chief Executive Editor of Frontiers. The authors have not responded to correspondence regarding this retraction.”

